# Relationship between Neuroimaging and Cognition in Frontotemporal Dementia: A [18 F]FDG PET and Structural MRI Study

**DOI:** 10.21203/rs.3.rs-3846125/v1

**Published:** 2024-01-15

**Authors:** Salih Cayir, Tommaso Volpi, Takuya Toyonaga, Jean-Dominique Gallezot, Yang Yanghong, Faranak Ebrahimian Sadabad, Tim Mulnix, Adam P. Mecca, Arman Fesharaki-Zadeh, David Matuskey

**Affiliations:** Yale University School of Medicine; Yale University School of Medicine; Yale University School of Medicine; Yale University School of Medicine; Yale University School of Medicine; Yale University School of Medicine; Yale University School of Medicine; Yale University School of Medicine; Yale University School of Medicine; Yale University School of Medicine

## Abstract

**Background:**

Frontotemporal dementia (FTD) is a clinically and pathologically heterogeneous condition with a prevalence comparable to Alzheimer’s Disease for patients under sixty-five years of age. Gray matter (GM) atrophy and glucose hypometabolism are important biomarkers for the diagnosis and evaluation of disease progression in FTD. However, limited studies have systematically examined the association between cognition and neuroimaging in FTD using different imaging modalities in the same patient group.

**Methods:**

We examined the association of cognition using Montreal Cognitive Assessment (MoCA) with both GM volume and glucose metabolism using structural magnetic resonance imaging (MRI) and ^18^F-fluorodeoxyglucose positron emission tomography scanning ([^18^F]FDG PET) in 21 patients diagnosed with FTD. Standardized uptake value ratio (*SUVR*) using the brainstem as a reference region was the primary outcome measure for [^18^F]FDG PET. Partial volume correction was applied to PET data to account for disease-related atrophy.

**Results:**

Significant positive associations were found between whole-cortex GM volume and MoCA scores (r = 0.461, p = 0.035). The association between whole-cortex [^18^F]FDG *SUVR* and MoCA scores was not Significant (r = 0.374, p = 0.094). GM volumes of the frontal cortex (r = 0.540, p = 0.011), caudate (r = 0.616, p = 0.002), and insula (r = 0.568, p = 0.007) were also Significantly correlated with MoCA, as were *SUVR* values of the insula (r = 0.508, p = 0.018), thalamus (r = 0.478, p = 0.028), and posterior cingulate cortex (PCC) (r = 0.472, p = 0.030).

**Discussion:**

Whole-cortex atrophy is associated with cognitive dysfunction, and this effect is larger than for cortical hypometabolism as measured with [^18^F]FDG PET. At the regional level, focal atrophy and/or hypometabolism in the frontal lobe, insula, PCC, thalamus, and caudate seem to imply the importance of these regions for the decline of cognitive function in FTD. Furthermore, these results highlight how functional and structural changes may not overlap and might contribute to cognitive dysfunction in FTD in different ways. Our findings provide insight into the relationships between structural, metabolic, and cognitive changes due to FTD.

## Introduction

Frontotemporal dementia (FTD) is an umbrella term for clinically, genetically, and pathologically heterogeneous neurodegenerative conditions associated with focal neuronal loss in the frontal and temporal lobes (Gorno-Tempini et al., 2011; Rascovsky et al., 2011). Behavioral variant FTD (bvFTD) is the most common subtype, encompassing more than half of all FTD cases with prominent executive function deficits, apathy, and/or disinhibition along with cognitive decline over the disease progression (Johnson et al., 2005; Rascovsky et al., 2011). Semantic variant primary progressive aphasia (svPPA) and non-fluent variant primary progressive aphasia (nfvPPA) are language subtypes of FTD with early manifestations of progressive decline in language abilities (Gorno-Tempini et al., 2011; Hodges & Patterson, 2007). Some patients may have clinical features of both FTD and motor neuron disease (MND), leading to a syndromic diagnosis of FTD-MND (Strong et al., 2009). In terms of the underlying neuropathology, FTD is mainly associated with pathological accumulation of TAR-DNA-binding protein-43 (TDP-43; FTD-TDP) or microtubule-associated protein tau (FTD-tau) and, less commonly, fused in sarcoma (FTD-FUS) (van der Ende & van Swieten, 2021).

^18^F-fluorodeoxyglucose positron emission tomography scanning ([^18^F]FDG PET) and structural magnetic resonance imaging (MRI) are essential tools for studying disease progression in FTD and moving the diagnosis from ‘possible’ to ‘probable’ bvFTD (Gorno-Tempini et al., 2011; Rascovsky et al., 2011; Ward et al., 2023). Moreover, predominant frontal and anterior temporal lobe atrophy and similar but more widespread hypometabolism are accepted as the core neuroimaging feature of FTD patients for clinical diagnosis (S. I. Dev et al., 2021; Howard J Rosen et al., 2002). Limbic and paralimbic areas—namely the anterior insula, amygdala, and anterior cingulate cortex (ACC), as well as the hippocampus, caudate, and thalamus—are also reported to be affected in patients with FTD (Sheena I. Dev et al., 2021; Franceschi et al., 2005; Seeley et al., 2008). The evolution of atrophy and hypometabolism beyond these core regions into the anterior parietal cortex and posterior cingulate cortex (PCC) might be seen as the disease progresses (Bejanin et al., 2020; Diehl-Schmid et al., 2007). However, these neuroimaging biomarker patterns might vary depending on the clinical and pathological subtypes of FTD. Specifically, atrophy/hypometabolism is more prominent in the left posterior fronto-insular cortex (Howard J Rosen et al., 2002) in cases of nfvPPA, in the anterior temporal lobes (generally asymmetric) (Howard J Rosen et al., 2002) in cases of svPPA, and in the frontal and anterior temporal cortices in cases of bvFTD (H. J. Rosen et al., 2002). Lastly, although these functional and structural changes in FTD overlap most of the time (Ward et al., 2023), a mismatch within brain regions can exist (Bejanin et al., 2020; Buhour et al., 2017; Ward et al., 2023; Woost et al., 2013).

Progressive global decline in cognitive function is a challenging and important symptom in all patients diagnosed with any type of dementia (Petersen, 2011), including FTD (Staffaroni et al., 2019). Moreover, primary deficits in executive function, with relatively spared memory abilities, are suggested as key diagnostic symptoms forming the 2011 International Consensus diagnostic criteria for bvFTD (Rascovsky et al., 2011). Literature connecting cognitive impairment to metabolic loss in FTD is relatively sparse, with only a few studies utilizing multiple imaging modalities (MRI and [^18^F]FDG PET) in the same patients with FTD (Chu et al., 2021; Kanda et al., 2008; Rajagopalan & Pioro, 2019; Woost et al., 2013). Importantly, in most studies (except (Woost et al., 2013)), [^18^F]FDG PET data were not corrected for partial volume effects, which may cause biased results due to the impact of GM atrophy in FTD (Quarantelli et al., 2004; Rousset et al., 1998). Also, in these studies, the primary measure of cognitive function was the Mini-Mental State Examination (MMSE), which has shown limited effectiveness in detecting cognitive impairments associated with FTD compared to the Montreal Cognitive Assessment (MoCA) (Deutsch et al., 2016). The MoCA includes specific items that assess frontal lobe processing, enhancing its sensitivity to detect frontal lobe dysfunction compared to the MMSE (Julayanont et al., 2014).

Thus the primary aim of this retrospective cross-sectional study is to investigate associations between cognitive impairment–as measured with MoCA– and brain structural and functional changes in FTD, as measured by T1-weighted (T1W) MRI and partial volume corrected (PVC) [^18^F]FDG PET, respectively. Additionally, in a subgroup of ten patients, cerebrospinal fluid (CSF) total tau (t-tau) levels (which are associated with the intensity of neuronal damage in neurodegeneration (Schraen-Maschke et al., 2008)) were correlated with neuroimaging results to explore whether neuroimaging biomarkers reflect CSF neuropathological changes.

## Methods

### Participants

We conducted a retrospective cross-sectional study with participants selected from records of patients seen at Yale New Haven Hospital’s (YNHH) Memory Clinic in CT, USA, covering the period from 2015 to 2023. All data were retrieved from electronic medical files. Patients were included if they met the following criteria: (1) diagnosis of FTD, including the FTD subtypes of bvFTD, nfvPPA, svPPA, and FTD-MND, according to consensus criteria and based on prior clinical assessment and available clinical information (Gorno-Tempini et al., 2011; Rascovsky et al., 2011; Strong et al., 2009); (2) inpatient or outpatient visit for evaluation by a cognitive disorders specialist at the YNHH Memory Clinic; (3) both 3D volumetric T1-weighted MRI and [^18^F]FDG PET scans; (4) assessed with the MoCA within three months of [^18^F]FDG PET. The main exclusion criteria were a history of another severe neurological or psychiatric disorder that could affect cognitive functioning, such as alcohol use disorder or cerebrovascular disease. Patients with evidence of a large cerebral mass, infarction, and/or hemorrhage in their neuroimaging data were also excluded. Additionally, patients with technical scan issues, such as MRI motion artifacts, were excluded. Twenty-one patients with FTD were included and further classified according to disease subtypes as bvFTD (n = 14), FTD-PPA (n = 6), and FTD-MND (n = 1). To increase power in the correlation analyses, all FTD subtypes were combined, but data for each subgroup were reported in the supplementary material (Table S1).

### Demographic and Clinical Variables

MoCA scores, available CSF results (n = 10), and demographic information, including age, sex, years of education, and months since disease onset, were extracted from each patient’s chart. Our database includes item-level data for MoCA scores; hence, we calculated the index scores (Memory, executive function, visuospatial function, language, attention, orientation) based on validated methods reported previously(Julayanont et al., 2014). A lumbar puncture was performed on ten patients by YNHH neurologists. All collected CSF samples were sent to commercial laboratories (Athena Diagnostics or Mayo Clinic) for core AD biomarker analysis, including t-tau (https://www.athenadiagnostics.com/view-full-catalog/a/admark-reg;-alzheimer-s-evaluation and https://www.mayocliniclabs.com/test-catalog/overview/607273).

### Magnetic resonance imaging and analysis

All MRI scans were performed at YNHH. T1W MPRAGE MR images of all participants were acquired on a 3T MRI scanner (Siemens, Verio). T1W MR images were skull- and muscle-stripped using the Computational Anatomy Toolbox for the Analysis of Structural MRI Data (CAT12, https://neuro-jena.github.io/cat/). FreeSurfer version (6.0.0) (Fischl, 2012) was used to create a segmented label map for each subject. To reduce the number of examined regions and minimize multiple comparisons, the main analysis was performed by combining all cortical regions into a whole-cortex ROI. Volumetric analysis of the frontal, temporal, parietal, and occipital cortex, ACC, PCC, insula, thalamus, amygdala, caudate, putamen, and hippocampus were also performed. These regions were selected based on their involvement in FTD (Staffaroni et al., 2019) and average bilateral values were used.

### [^18^F]FDG PET imaging and analysis

All PET scans were conducted at YNHH using either a Discovery PET/computed tomography (CT) system (GE Healthcare, n = 17) or Siemens Verio (Siemens Medical Solutions, n = 4). All patients underwent whole-body PET examinations at rest after intravenous injection of an average of 10.1 mCi (range: 9.2 to 11.1) [^18^F]FDG. PET data were acquired for 10 minutes starting at approximately 50 minutes post-injection.

The static PET image (50–60 min) was aligned to each subject’s MR image via rigid registration. Regional values were obtained by transforming the FreeSurfer-labeled ROIs from MR to PET space. The same regions already mentioned for MRI were analyzed (average bilateral values).

The extracted region-wise PET values ([Bq/mL]) were normalized into standardized uptake value ratios (*SUVR*, [unitless]) using the brainstem ROI as a reference region, as in previous work (Beyer et al., 2021; Dukart et al., 2013). To account for potential partial volume effects due to atrophy, PVC was performed on [^18^F]FDG PET images using the Iterative Yang method(Erlandsson et al., 2012), as we have previously described (Lu et al., 2021). A 5-mm full-width half maximum (FWHM) Gaussian kernel was used as point spread function (PSF) for both the Siemens Verio and GE Discovery scanners.

### Statistical analysis

After the normality of variables was assessed using the Kolmogorov–Smirnov test (p > 0.05), unpaired t-tests (significance level alpha = 0.05) were used to examine differences in continuous variables between the bvFTD and FTD-PPA groups, and the chi-square test (alpha = 0.05) was employed for categorical variables. Relationships between neuroimaging (whole-cortex and regional GM volume, SUVR), CSF (t-tau), MoCA scores and demographic information (age, years of education, and duration of symptoms) were assessed using Pearson’s correlation (two-tailed, alpha = 0.05). P-values were not corrected for multiple comparisons, considering the exploratory nature of the study. Statistical analysis was performed with MATLAB (2023a, The MathWorks, Inc.), and GraphPad Prism (v. 9.0).

## Results

### Demographic and clinical characteristics

Demographic and clinical information are presented in Table 1. Detailed reports for MoCA index scores, and neuroimaging data across different FTD subtypes are provided in the Supplementary Materials (Supplementary Table S1A, S1B). There were no significant correlations between MoCA scores and age (r = 0.39, p = 0.07), years of education (r = 0.05, p = 0.82), or duration of symptoms (r = 0.24, p = 0.28). As expected, patients with FTD-PPA had significantly lower MoCA language index scores (MoCA-LIS) than the bvFTD group (p = 0.046) (Table S1A).

**Figure 1:**
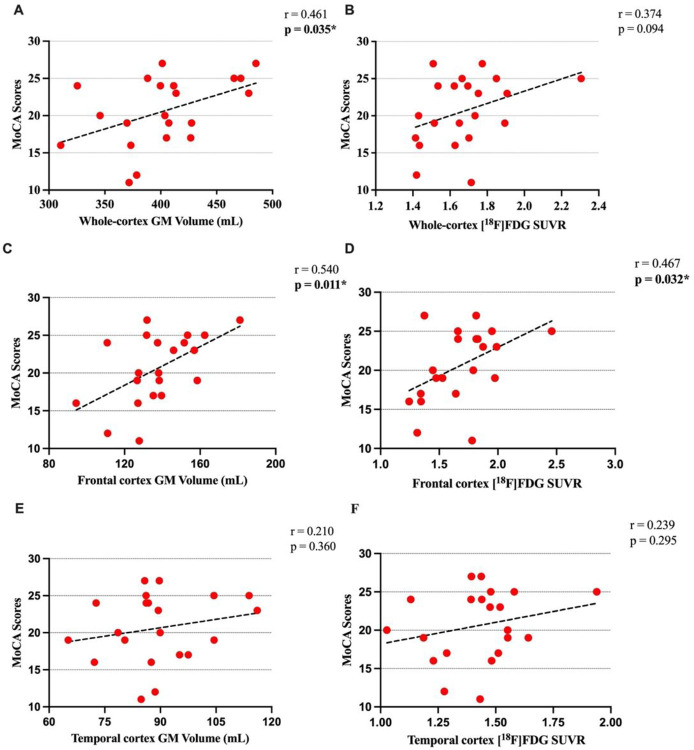
Scatterplots of the associations of Montreal Cognitive Assessment (MoCA) scores with cortical GM volume (A, C, E) and [^18^F]FDG SUVR (B, D, F); *: Pearson’s correlation results are significant (*) at alpha = 0.05 level (uncorrected).

**Figure 2:**
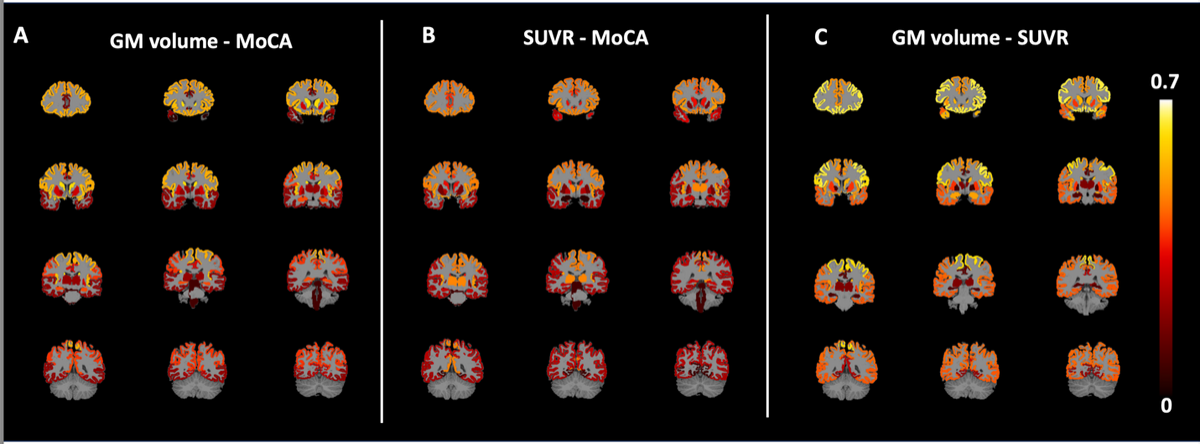
Visual representation of the associations (Pearson’s r) between region-wise GM volume and MoCA scores (A), region-wise [^18^F]FDG SUVR and MoCA scores (B), region-wise GM volume and [^18^F]FDG SUVR (C). The same value for left and right GM volume and SUVR is shown for each Freesurfer ROI. Significance of correlations is not highlighted.

There was a significant positive association between whole-cortex GM volume and MoCA scores (r = 0.461, p = 0.035, Fig. 1A). As an exploratory analysis, we investigated the associations between GM volumes of different ROIs and MoCA scores (Table 2; Fig. 1C, E; Fig. 2A). GM volume had a significant positive association with MoCA in the frontal cortex (r = 0.540, p = 0.011), insula (r = 0.568, p = 0.007), and caudate (r = 0.616, p = 0.002) (Table 2). We also explored the associations between cortical GM volume and MoCA cognitive domain index scores. Cortical GM volume had a significant positive association only with the executive index scores (Supplementary Table 2). At the regional level, GM volumes of the frontal, parietal cortex, caudate, and insula were significantly associated with executive index scores, and GM volume of the temporal lobe was significantly associated with visuospatial index scores. Also, there was a positive trend of association between GM volumes of the hippocampus and memory index scores (r = 0.423, p = 0.055) (Fig. S1A).

### Correlations between brain metabolism and cognition in FTD

The positive association between whole-cortex *SUVR* values and MoCA scores was not significant (r = 0.374, p = 0.094, Fig. 1B). Like the voxel-based results, we investigated the associations between *SUVR* of different ROIs and MoCA scores (Table 2; Fig. 1D, F; Fig. 2B). Regional *SUVR* had a significant positive association with MoCA scores in the frontal cortex (r = 0.467, p = 0.032), posterior cingulate cortex (r = 0.472, p = 0.030), insular cortex (r = 0.508, p = 0.018) and thalamus (r = 0.478, p = 0.028) (Table 2).

We additionally explored the associations between whole-cortex *SUVR* values and specific MoCA cognitive domain index scores. Whole-cortex *SUVR* values had no significant associations with any of the MOCA domains (Supplementary Table 2).

### Correlations between brain metabolism and GM volume in FTD

When examining the association between whole-cortex GM volume and *SUVR*, we found a significant positive correlation (r = 0.506, p = 0.02). As shown in Fig. 2C, there was also a significant positive correlation between GM volume and *SUVR* in frontal (r = 0.615, p = 0.003) and parietal (r = 0.437, p = 0.047) cortices, caudate (r = 0.545, p = 0.010), insula (r = 0.502, p = 0.020), ACC (r = 0.487, p = 0.025), and amygdala (r = 0.522, p = 0.015). However, no significant correlations were observed in the temporal cortex (r = 0.419, p = 0.059), occipital cortex (r = 0.211, p = 0.357), thalamus (r = 0.177, p = 0.443), hippocampus (r = 0.104, p = 0.654), PCC (r = 0.26, p = 0.254), or putamen (r = 0.383, p = 0.087).

### Correlations between t-tau levels and neuroimaging results in FTD

**Figure 3:**
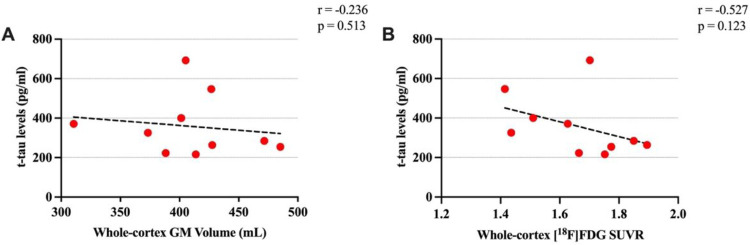
Scatterplots of the associations of total tau (t-tau) levels with whole-cortex GM volume (A) and whole-cortex [18F]FDG SUVR (B). Pearson’s correlation results are significant (*) at alpha = 0.05 level (uncorrected).

Figure 3 illustrates the correlations between neuroimaging data and t-tau levels among the patients with available CSF data (n = 10). There was a non-significant negative association between t-tau levels and both cortical GM volume (r=−0.23, p = 0.51) and *SUVR* (r=−0.53, p = 0.12) (Fig. 3B).

## Discussion

In this study, we investigated the association between cognitive impairment, cortical atrophy, and hypometabolism in FTD. Additionally, we explored the associations between global or domain-specific cognitive performance and previously reported cortical and subcortical regions that are affected in FTD.

Cortical atrophy has been suggested as an important neurobiological correlate of cognitive decline in various neurodegenerative diseases, including FTD (Hartikainen et al., 2012; Mesulam et al., 2014; Möller et al., 2015; Nicastro et al., 2020). Consistent with the literature, our findings indicate that whole-cortex atrophy is significantly associated with worse global cognitive performance in patients with FTD. When examining individual cognitive domains, this association was particularly robust with executive function.

This finding is consistent with the fact that decline in executive ability is accepted as one of the defining symptoms of early FTD (Rascovsky et al., 2011). Moreover, apathy–a core behavioral symptom of FTD along with disinhibition– has been shown to be associated with executive dysfunction and to affect similar cortical regions according to imaging studies (Ducharme et al., 2018; Eslinger et al., 2012; Malpetti et al., 2021). While other cognitive domains show positive trends of association with global cortical GM volume, none of these reaches significance. Notably, the association between whole-cortex GM volume and the memory domain appears to be the weakest among all cognitive domains (r = 0.10, p = 0.67). This may be partly attributed to the fact that patients are in a relatively early stage of the disease (disease duration: 30.6 ± 17.8 months), which is consistent with the expectation of preserved memory function in early FTD (Bott et al., 2014). This is also supported by a recent study by Ang et al., in a large cohort of patients with FTD (n = 602) demonstrated how the MoCA memory domain is relatively ineffective in distinguishing FTD from healthy controls as compared to executive and language domains (Ang et al., 2023). Of note, the lack of significant results for the language domains is likely to be explained by the predominant diagnosis of probable bvFTD in the study cohort (n = 14), with a smaller number diagnosed with FTD-PPA (n = 6).

Whole cortex hypometabolism was not significantly associated with global cognitive function or any individual cognitive domain. The lack of Significant results with whole-cortex [18F]FDG PET uptake seems surprising, given that hypometabolism is expected to precede volume loss, and progress faster and in a more widespread fashion in FTD, according to a recent review (Ward et al., 2023) and multiple studies (Bejanin et al., 2020; Jeong et al., 2005; Kanda et al., 2008). However, when examining FTD-affected regions, in contrast to the whole-cortex, significant associations between poor cognitive performance and hypometabolism emerged in the frontal cortex, PCC, thalamus, and insula. These findings might be explained by the focal rather than global role of hypometabolism in modulating cognitive dysfunction. A similar but higher degree of association was also observed between GM atrophy and cognitive impairment in the frontal cortex and insula. Consistent with our findings, the role of the frontal cortex in cognition has been well established in FTD patients in studies using [^18^F]FDG PET and structural MRI (Ferreira et al., 2016; Jobson et al., 2021; Kanda et al., 2008; Woost et al., 2013). Indeed, frontal cortical atrophy and hypometabolism are the core neuroimaging findings across different subtypes of FTD and are responsible for the unique characteristics of the disease, such as executive dysfunction (prefrontal cortex (Sheena I. Dev et al., 2021)) and agrammatism (left inferior frontal cortex (Rohrer et al., 2009)) for bvFTD and nfvPPA, respectively. The insular cortex has been proposed to be a central hub for different brain networks responsible for a wide range of cognitive functions and the earliest affected region in FTD (Seeley, 2010). This crucial role of the insula is also shown in patients with FTD in an extensive meta-analysis by Fathy et al. (Fathy et al., 2020) and two different longitudinal studies (Bejanin et al., 2020; Rajagopalan & Pioro, 2019).

Interestingly, cognitive performance was significantly associated only with metabolic changes (not GM volume loss) in the thalamus and PCC. In a study conducted on large cohort of bvFTD patients by Vuksanovic et al., the thalamus was found to be consistently affected by atrophy in bvFTD, as part of the dorsolateral-prefrontal and orbitofrontal circuits related to executive function, motor programming, personality and mood (Vuksanović et al., 2021). Thalamic atrophy is also particularly prominent in FTD cases with TDP-43 pathology and C9orf72 mutations (Bocchetta et al., 2018). Moreover, a relationship between decreased PCC glucose metabolism and memory function has been shown in patients with FTD (Scheltens et al., 2018). In the current study, PCC hypometabolism was associated not only with the memory domain but also with global cognitive performance, and this requires further investigation on a larger cohort. We also found a significant association between caudate volume loss and cognitive performance. It is not surprising to find caudate atrophy related to cognitive impairment, considering the growing body of evidence on the role of the striatum in cognitive function as part of the fronto-striatal circuit (Koziol & Budding, 2020; Morris et al., 2016). The caudate has also been shown to have a relatively high degree of synaptic loss in normal aging, as compared to the rest of the brain (Toyonaga et al., 2023). As to FTD patients, Macfarlane et al. have linked worse cognitive performance with a higher degree of caudate atrophy (Macfarlane et al., 2015), while Looi et al. found significant associations between left caudate volume and MMSE scores of patients with FTD (Looi et al., 2008).

We did not detect any significant association between cognitive impairment and neuroimaging variables in the temporal, parietal and occipital cortex, ACC, putamen, amygdala, or hippocampus. Among these regions, non-significant results in the temporal cortex warrant further discussion, given its well-established role in FTD pathology as demonstrated in various longitudinal neuroimaging studies (Diehl-Schmid et al., 2007; Howard J Rosen et al., 2002; Staffaroni et al., 2019). The heterogeneity of our study population might be part of the explanation. We had six patients diagnosed with FTD-PPA, and only three of them were specifically svPPA. Peak atrophy at baseline in svPPA is reported to be temporal cortex dominant, and the rate of temporal GM and metabolic loss is the fastest (3–4% per six months for GM) among all other FTD variants (Sheena I. Dev et al., 2021). Further studies including more patients with svPPA diagnosis will be crucial to examine the association between temporal cortical structure/function alterations and cognition in FTD.

We also examined the association between CSF levels of t-tau and neuroimaging biomarkers in a subgroup of ten patients, and we found a negative trend of association between CSF tau levels and both global cortical metabolism and GM volume. Supporting our findings, two previous studies showed more FTD and AD-like [^18^F]FDG patterns –hypometabolic areas around frontal, temporal, and parietal cortices–in CSF t-tau positive groups than in t-tau negative groups (Caminiti et al., 2018; Cerami et al., 2015). Interestingly, a study by Fenu et al. could not find a significant association between whole brain GM volume and t-tau levels in patients with FTD (Fenu et al., 2022). Our data suggest that CSF findings, which are expected to reflect neuronal degeneration, may agree with [^18^F]FDG PET more than with structural MRI results. However, more studies in a larger population are necessary to make a conclusive statement.

Global and local (ROI-specific) discordance between structural and metabolic changes in their relationship with cognitive function needs to be addressed. Incongruence between GM atrophy and functional changes has been suggested previously in different types of dementias (Rodriguez-Oroz et al., 2015) as well as in normal aging (Toyonaga et al., 2023). In a study conducted by Shimizu et al. to examine discordance between brain perfusion and atrophy in FTD, the authors have shown that atrophy might exist in the presence of normal metabolism in some regions, whereas hypometabolism can exceed the atrophy in other cortical regions (Shimizu et al., 2010). A longitudinal study by Bejanin et al. showed that progression in hypometabolism and atrophy was unique to each brain region in FTD (Bejanin et al., 2020). Cross-sectional studies have shown similar results(Kanda et al., 2008; Woost et al., 2013). Consistent with the literature, we also found mild to moderate significant correlations between GM atrophy and hypometabolism in frontal and parietal cortices, caudate, insula, ACC, and amygdala, but non-significant associations in the remaining regions. This structure/function mismatch helps to understand why fewer significant associations between hypometabolism and cognition were detected with respect to GM atrophy vs. cognition. Additionally, [^18^F]FDG PET measures might have intrinsically limited sensitivity to explain the cognitive impairment. Studies by Jacova et al. 2013 and Clarke et al. 2021 show anterior brain hypometabolism among the asymptomatic FTD mutation carriers as compared to normal controls (Clarke et al., 2021; Jacova et al., 2013). Also, in a study examining the biomarker value of [^18^F]FDG PET in FTD, the presence of hypometabolism has been found to have a sensitivity of 47% to predict cognitive decline over two years (Kerklaan et al., 2014). An ideal neuroimaging biomarker for FTD seems to be still missing, and there is a growing interest in a more accurate disease marker than hypometabolism in FTD, like synaptic density markers (e.g., [^11^C]UCB-J) (Carson et al., 2022).

The main limitation is the small number of patients in each FTD subgroup, which prevented us from carrying out separate analyses for bvFTD, svPPA, and nfvPPA. Other limitations include the lack of histopathological confirmation of diagnosis, and use of MoCA as the only measure of cognitive functioning without a more comprehensive cognitive assessment. However, MoCA is a well validated screening tool with enhanced sensitivity to frontal lobe dysfunction (e.g., “ executive functioning”, “phonemic fluency,” and “abstraction”), with respect to MMSE, which is particularly relevant for patients with FTD (Ang et al., 2023). Finally, our FDG-PET data originates from two different scanners (GE Discovery, n = 17; Siemens Verio, n = 4), which may produce some inconsistency in the data. However, we were able to detect significant associations with biological plausibility and the use of different scanners reflects real-world circumstances common in clinical settings.

## Conclusion

Overall, our data suggest that global cortical atrophy, and not hypometabolism as measured with [^18^F]FDG PET, is associated with cognitive dysfunction. Also, on a regional level, focal atrophy and/or hypometabolism of the frontal lobe, insula, PCC, thalamus, and caudate emerge as important for the decline in cognitive function in FTD. These measures may not overlap in their association of cognitive function and might present with unique patterns. Future studies examining the association between cognition and neuroimaging measures in FTD are necessary. These studies should utilize different modalities simultaneously, incorporate larger sample sizes, and include subgroup-specific analyses.

## Figures and Tables

**Table 1:** Patient demographics and clinical characteristics

	FTD (n = 21)
Age	69.3 ± 8.1
Sex (male/female)	16/5
Education (years)	15.8 ± 2.6
Disease Duration (months)	30.6 ± 17.8
MoCA	20.6 ± 4.6
t-tau (pg/ml) (n = 10*)	357.6 ± 154.1
Interval between MoCA and PET scan (days)	41.1 ± 35.8

Abbreviations: MoCA: Montreal Cognitive Assessment, t-Tau; Total Tau.

Notes: mean ± SD of each variable are reported.

* n = 21 for all variables except t-Tau (n = 10)

**Table 2:** Regional correlation of GM volume and [18F]FDG SUVR with MoCA scores

	GM volume		[^18^F]FDG SUVR	
Regions	r	p	r	p
Whole cortex	0.461	0.035*	0.374	0.094
Frontal cortex	0.540	0.011*	0.467	0.032*
Temporal cortex	0.210	0.360	0.239	0.295
Parietal cortex	0.384	0.085	0.269	0.238
Occipital cortex	0.266	0.242	0.068	0.769
Anterior cingulate cortex	0.172	0.455	0.380	0.089
Posterior cingulate cortex	0.401	0.071	0.472	0.030*
Insula	0.568	0.007**	0.508	0.018*
Thalamus	0.210	0.358	0.478	0.028*
Amygdala	0.218	0.340	0.040	0.860
Caudate	0.616	0.002**	0.346	0.123
Putamen	0.302	0.183	0.162	0.482
Hippocampus	0.406	0.067	0.282	0.214

MoCA: Montreal Cognitive Assessment; GM: Gray matter; FTD: Frontotemporal Dementia; SUVR:

Standardized uptake value ratio

*: Results are Significant at alpha = 0.05 level (uncorrected).

**: Results are Significant at alpha = 0.01 level (uncorrected).

## Data Availability

The datasets used and/or analyzed during the current study are available from the corresponding author on reasonable request.
